# High-Efficiency Diphenylpyrimidine Derivatives Blue Thermally Activated Delayed Fluorescence Organic Light-Emitting Diodes

**DOI:** 10.3389/fchem.2020.00356

**Published:** 2020-05-14

**Authors:** Sunyoung Sohn, Min Woo Ha, Jiyong Park, Yoo-Heon Kim, Hyungju Ahn, Sungjune Jung, Soon-Ki Kwon, Yun-Hi Kim

**Affiliations:** ^1^Department of Creative IT Engineering, Pohang University of Science and Technology, Pohang-si, South Korea; ^2^Department of Chemistry and Research Institute for Green Energy Convergence Technology, Gyeongsang National University, Jinju-si, South Korea; ^3^Center for Catalytic Hydrocarbon Functionalizations, Institute for Basic Science (IBS), Daejeon, South Korea; ^4^Department of Chemistry, Korea Advanced Institute of Science and Technology (KAIST), Daejeon, South Korea; ^5^Pohang Accelerator Laboratory, Pohang-si, South Korea; ^6^Department of Materials Engineering and Convergence Technology and ERI, Gyeongsang National University, Jinju-si, South Korea

**Keywords:** organic light-emitting diode, thermally activated delayed fluorescence, blue emitter, diphenylpyrimidine, singlet–triplet energy gap

## Abstract

Organic light-emitting diodes with thermally activated delayed fluorescence emitter have been developed with highly twisted donor–acceptor configurations and color-pure blue emitters. Synthesized 4-(4-(4,6-diphenylpyrimidin-2-yl)phenyl)-10H-spiro[acridine-9,9′-fluorene] (4,6-PhPMAF) doped device with spiroacridine as a donor unit and diphenylpyrimidine as acceptor exhibits the device characteristics such as the luminescence, external quantum efficiencies, current efficiencies, and power efficiencies corresponding to 213 cd/m^2^, 2.95%, 3.27 cd/A, and 2.94 lm/W with Commission International de l'Eclairage (CIE) coordinates of (0.15, 0.11) in 4,6-PhPMAF-doped DPEPO emitter. The reported 10-(4-(2,6-diphenylpyrimidin-4-yl)phenyl)-10H-spiro[acridine-9,9′-fluorene] (2,6-PhPMAF) doped device exhibit high device performance with 1,445 cd/m^2^, 12.38%, 19.6 cd/A, and 15.4 lm/W, which might be originated from increased internal quantum efficiency by up-converted triplet excitons to the singlet state with relatively smaller Δ*E*_ST_ of 0.17 eV and higher reverse intersystem crossing rate (*k*_RISC_) of 1.0 ×10^8^/s in 2,6-PhPMAF than 0.27 eV and 3.9 ×10^7^/s in 4,6-PhPMAF. Despite low performance of 4,6-PhPMAF doped device, synthesized 4,6-PhPMAF has better color purity as a deep-blue emission with *y* axis (0.11) than reported 2,6-PhPMAF with *y* axis (0.19) in CIE coordinate. The synthesized 4,6-PhPMAF has higher thermal stability of any transition up to 300°C and decomposition temperature with only 5% weight loss in 400°C than reported 2,6-PhPMAF. The maximum photoluminescence emission of 4,6-PhPMAF in various solvents appeared at 438 nm, which has blue shift about 20 nm than that of 2,6-PhPMAF, which contributes deep-blue emission in synthesized 4,6-PhPMAF.

## Introduction

The organic light-emitting diodes (OLEDs) using thermally activated delayed fluorescence (TADF) material have been widely investigated for high-efficiency device performance or low triplet and singlet energy levels for reduced driving voltage by narrow host bandgap, since the Adachi group reported the intersystem crossing (ISC) and reverse intersystem crossing (RISC) for triplet-to-singlet state conversion (Goushi et al., 2012; Nakanotani et al., 2014; Kim et al., 2015). It can harvest both single and triplet excitons because TADF involves small singlet–triplet state energy splitting by thermally activation (Tao et al., 2014; Sun et al., 2016; Cui et al., 2017). Improvement in device efficiency has been reported for device based on TADF molecules that the azasiline donor unit-based intermolecular charge-transfer exhibited deep blue TADF dye of high efficiency of 22.3% in mixed co-host (Kwon et al., 2015), and the methoxy substituents to replace tert-butyl substituents on the carbazole donors have been found to decrease the singlet–triplet state energy splitting (Δ*E*_ST_), as well as long lifetime and reducing the device efficiency roll-off (Wu et al., 2014; Shizu et al., 2015). Triplet energy levels and reduction potentials of various acceptor cyano-substituted pyrazines were reported with combined small singlet–triplet splitting and large fluorescence rate (Liu et al., 2019). This molecular design can be accomplished with acridine, carbazole, and phenoxazine as electron donor units and/or sulfone, phosphine oxide, dimesitylboryl, and polycyclic borazine as electron acceptor units (Ganesan et al., 2018). Furthermore, as way of improving efficiency by device engineering, introducing the dual delayed fluorescence in the host material exhibited the slow-efficiency roll-offs (Zhang et al., 2019). Another device engineering technique involves measuring transition dipole moment, where the emitter with planar molecular structure becomes horizontally orientated, resulting in improved light-outcoupling efficiency of more than 30% (Mayr et al., 2014; Komino et al., 2017). In other groups, highly ordered morphology and horizontal transition dipole moment ratio of Pt(II)-based [Pt(fppz)_2_] and spiro[acridine-9,9′-fluorene] donor with varied pyridyl orientation have been reported. High horizontal transition dipole moment ratio in para-linked donor between the donor–acceptor orientation has been measured using angle-dependent photoluminescence (PL) measurement (Ganesan et al., 2018). In our previous TADF work, we found that an *m*-phenyl linker between the electron-donating and the electron-accepting units showed higher device efficiencies; analysis of scattered X-ray intensities revealed a weak overlap in the phenyl linker, as well as well-aligned structure in the horizontal direction compared to those of the *p*-phenyl linker (Sohn et al., 2017). Based on the reported linker material and molecular orientation, we analyzed the properties of synthesized emitters with spiroacridine-based electron donor (D) and diphenylpyrimidine group of electron acceptor (A) with different substance nitrogenous position.

## Results and Discussion

### Synthesis and Characterization

The synthetic routes of 4,6-PhPMAF instead of 2,6-PhPMAF are shown in **Scheme S1**. The intermediate 10-(4-bromophenyl)-10*H*-spiro[acridine-9,9′-fluorene] and 2-chloro-4,6-diphenylpyrimidine were synthesized by Buchwald–Hartwig amination and Suzuki coupling reaction, respectively. The 4,6-PhPMAF was obtained by Suzuki coupling. The chemical structure of the synthesized intermediates and 4,6-PhPMAF were characterized by nuclear magnetic resonance (^1^H-NMR, ^13^C-NMR) spectroscopy and mass spectroscopy. Detailed synthesis and characterization procedures are described in the Supporting Information (Supplementary Figures 1, 2).

### Computational Analysis

**Figure 1:**
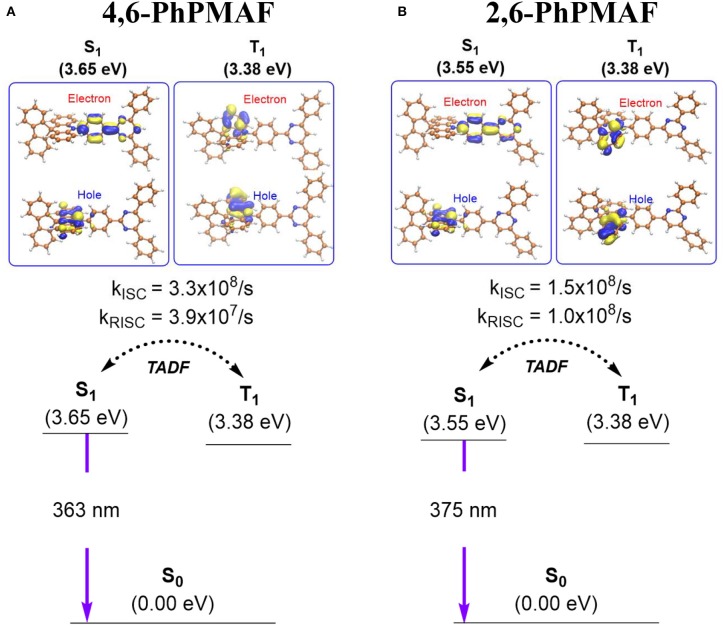
Summary of the computed excited state electronic structures and rates of intersystem crossing of **(A)** 4,6-PhPMAF and **(B)** 2,6-PhPMAF.

The ground state (*S*_o_) and the excited state electronic structures of 4,6-PhPMAF and 2,6-PhPMAF were compared by means of density functional theory (DFT). We located the *S*_o_ geometries using CAM-B3LYP/def2-SVP level of the theory (Supplementary Figure 3). The renditions of frontier molecular orbitals confirmed that the highest occupied molecular orientation (HOMO) levels of the two compounds are localized at the spiroacridinyl group, and the lowest unoccupied molecular orientation (LUMO) levels are present at the diphenyl pyrimidinyl group (Supplementary Figure 4). The computed HOMO and LUMO energy gaps of the two molecules were comparable: 5.61 eV for 4,6-PhPMAF and 5.70 eV for 2,6-PhPMAF. Figure 1 summarizes the computed excited state electronic structures of the first singlet excited state (*S*_1_) and the triplet excited state geometries (*T*_1_). The excited state geometries of the synthesized chromophores were optimized using a time-dependent DFT, namely, TD-CAM-B3LYP/def2-SVP level of the theory. The adiabatic electronic energies of the *S*_1_ states are 3.65 and 3.55 eV for 4,6-PhPMAF and 2,6-PhPMAF, respectively, relative to those of the optimized *S*_o_ geometries. The calculated vertical excitation energies of the *S*_1_ were 3.40 eV (363 nm) and 3.30 eV (375 nm) for 4,6-PhPMAF and 2,6-PhPMAF, respectively, which is in line with the observation that there is a blue shift in the photoluminescent spectrum of 4,6-PhPMAF with respect to that of 2,6-PhPMAF. The renditions of natural transition orbitals (NTOs) of the *S*_1_ states suggested the excited states bear charge transfer (CT) characters (Supplementary Figure 5): the hole (e^+^) is localized at the donor, and the electron (e^−^) is situated at the acceptor. The CT characters explained the solvatochromism observed experimentally (*vide infra*). The computed oscillator strength (*f* ) for analyzing the radiative transition from *S*_1_ and to *S*_o_ exhibits same scale at the order of 10^−3^ in 4,6-PhPMAF (*f* = 0.002640) or 2,6-PhPMAF (*f* = 0.004138). The dipole moment was measured to analyze the molecular orientation, as summarized in Supplementary Table 1. We also identified the triplet excited state geometries that are responsible for the observed TADF. The computed Δ*E*_ST_ were 0.27 and 0.17 eV for 4,6-PhPMAF and 2,6-PhPMAF, respectively. The visualizations of NTOs suggested the triplet excited states exhibit the characteristic of π-π^*^ transitions localized at the donor group (spiroacridine). We also computed the vibrational spin–orbit coupling (V_SOC_) strengths and the rates of ISC and RISC of the two molecules, as detailed in the supporting information. The *V*_SOC_ of two molecules IS obtained as 1.511 and 1.460 cm^−1^, as listed in Supplementary Table 2. The computed rates of ISCs explained the observed TADF behaviors (Figure 1 and Supplementary Table 2). For 4,6-PhPMAF, the computed rate of ISC (*k*_*ISC*_) was 3.3 ×10^8^/*s*, whereas that of the reverse ISC (*k*_*RISC*_) was 3.9 ×10^7^/*s*. For 2,6-PhPMAF, the computed rate of ISC (*k*_*ISC*_) was 1.5 ×10^8^/*s*, whereas that of the reverse ISC (*k*_*RISC*_) was 1.0 ×10^8^/*s*. Of note, the computed rates of RISC are significantly faster than the rates of phosphorescence quenching, which progresses in a time scale of microsecond or equivalently in a rate of 10^6^/*s*. Accordingly, the singlet and the triplet excited states are in equilibrium, and the fluorescence quenching from the *S*_1_ is the dominant channel of PL decaying. The PL quantum yield (PLQY) values of two molecules under excitation at 300 nm are determined to be 16.9% and 29.5%, as shown in Supplementary Table 2. It is measured by the absolute method using a Hamamatsu Quantaurus-QY. The luminescence quantum efficiencies are calculated by Quantaurus-QY Absolute PL quantum yield spectrometer (C11347-11). The maximum PL spectra of DPEPO:4,6-PhPMAF or DPEPO:2,6-PhPMAF in dilute oxygen-free chlorobenzene solution at low temperature are observed at 467.26 and 493.48 nm (Supplementary Figure 6).

### Thermal Properties

The thermal stability of 4,6-PhPMAF was studied by thermogravimetric analysis and differential scanning calorimetry. 4,6-PhPMAF did not show the any transition up to 300°C, and the decomposition temperature (*T*_d_), corresponding to 5% weight loss, was 400°C, whereas the reported 2,6-PhPMAF was observed in the glass transition at 158°C and *T*_d_ at 400°C. The results indicate that the 4,6-PhPMAF shows better thermal stability (Supplementary Figure 7 and Supplementary Table 3).

### Optical Properties

**Figure 2:**
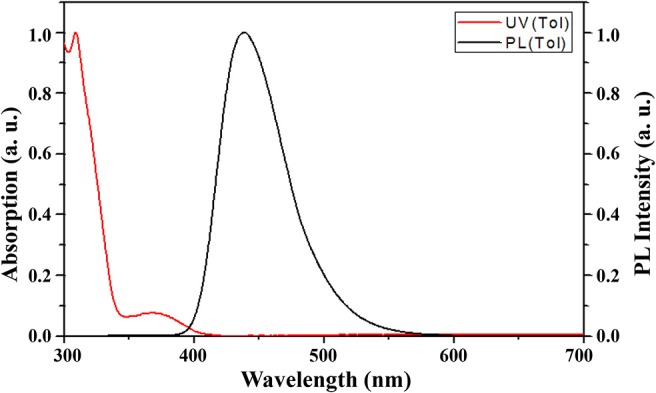
UV-visible and PL spectra of 4,6-PhPMAF in toluene solution.

The photophysical properties of 4,6-PhPMAF in various media are shown in Figure 2 and Supplementary Figure 8 and are summarized in Supplementary Table 2. The PL spectroscopy of various solution of 4,6-PhPMAF was measured in cyclohexane (ε = 2.02), toluene (ε = 2.38), chloroform (ε = 4.8), acetone (ε = 20.7), and DMF (ε = 36.7) (Supplementary Figure 8). 4,6-PhPMAF displayed strong solvatochromism with a red shift of its PL peak from 400 nm in cyclohexane to 530 nm in DMF, which indicate the charge transfer-type emission. On the contrary, the 2,6-PhPMAF shows stronger solvatochromism with red shift of its PL peak from 410 nm in cyclohexane to 560 nm, which is more than that of 4,6-PhPMAF. The absorbance and fluorescence spectra of 10^−5^ M 4,6-PhPMAF in toluene are depicted. The intense absorption in the range of 300–350 nm may be assigned to the absorption of *N*-phenyl-spiroacridine, whereas the relatively weak and broad absorption from 350 to 410 nm may be assigned to the intramolecular charge transfer excitation (Woo et al., 2019). The absorption behavior of 2,6-PhPMAF was similar to that of 4,6-PhPMAF). The UV-visible (UV-vis) onset of 4,6-PhPMAF and 2,6-PhPMAF was 3.03 and 2.95 eV, respectively. The maximum PL emission of 4,6-PhPMAF appeared at 438 nm, which was 20 nm blue shifted than that of 2,6-PhPMAF. From the results, it is expected that the color purity of new synthesized 4,6-PhPMAF will be better than that of reported 2,6-PhPMAF.

### Electrochemical Properties

The HOMO level of 4,6-PhPMAF was determined as −5.30 eV from the cyclovoltametric measurement, and LUMO level was determined as −2.27 eV by adding the optical bandgap (3.03 eV), whereas the HOMO and LUMO levels of 2,6-PhPMAF were determined as −5.26 and −2.31 eV by adding the optical bandgap (2.95 eV) (Supplementary Figure 9).

### Structural Properties

**Figure 3:**
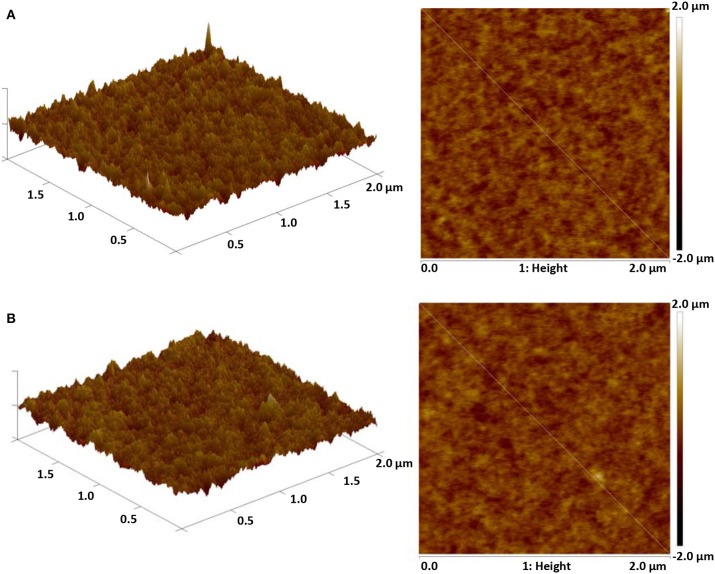
Three- and two-dimensional AFM topographical images of **(A)** DPEPO:4,6-PhPMAF and **(B)** DPEPO:2,6-PhPMAF of 2 ×2-μm scan size.

**Figure 4:**
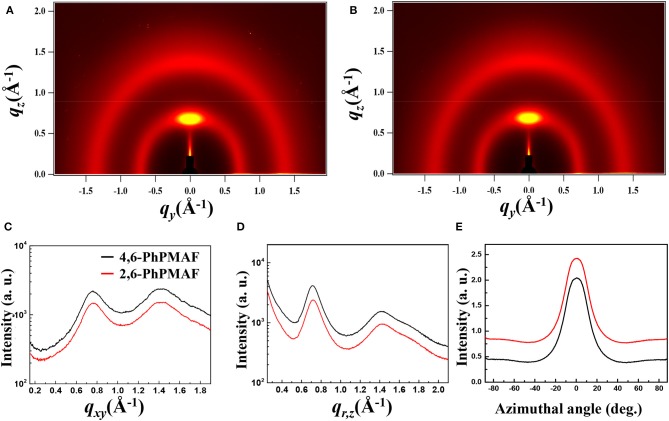
Two-dimensional grazing-incident wide-angle X-ray diffraction (2D GI-WAXD) images of scattered X-ray intensity from surface to full depth for films of **(A)** DPEPO:4,6-PhPMAF and **(B)** DPEPO:2,6-PhPMAF on SiO_2_/Si substrates, y axis: in-plane scattering; x axis: out-of-plane scattering. **(C)** In-plane scattering spectra; **(D)** out-of-plane scattering spectra. **(E)** Azimuthal intensity plots of orientation distributions of sets of crystallographic reciprocal lattice planes of the films.

The synthesized dopant 4,6-PhPMAF and 2,6-PhPMAF will be denoted as 1 and 2 from these results. The topographical three- and two-dimensional (2D) images of 22 wt.% 1 and 2 doped DPEPO emitters with thicknesses of 50 nm are measured using atomic force microscopy (AFM) analysis with 2 ×2-μm scan size, as shown in Figure 3. The root-mean-square (RMS) surface roughness (*R*_q_), average surface roughness (*R*_a_), and RMS roughness values of DPEPO:4,6-PhPMAF film in Figure 3A show uniform surface morphology of 0.2, 0.16, and 0.18 nm, respectively, and the DPEPO:2,6-PhPMAF film in Figure 3B shows 0.193, 0.153, and 0.15 nm, respectively. All films exhibit very uniform surfaces with a roughness value <0.2 nm due to strong π-π stacking with closely packed structures that yield good electrical device properties (Sohn et al., 2017). In order to precisely analyze the structural property of the emitters, we measured the two-dimensional grazing-incident wide-angle X-ray diffraction (2D GI-WAXD) to characterize the molecular orientation, as well as the packing properties of the emitters (Figure 4). In the patterns, *q*_*xy*_ and *q*_*z*_ represent the in-plane and out-of-plane components of the scattering vector *q*, which are normal to the plane of incidence and the film surface plane. The azimuthal intensity curve is measured to analyze the plots of orientation distribution. All films present similar distributions with a strong and broad diffraction peak, which might be attributed to the planar packing of random orientated emitters along the out-of-plane direction at *q*_*z*_ = 0.75 Å^−1^ in Figures 4A,B. To compare the diffraction peak position and crystallographic property, the in-plane (Figure 4C) and out-of-plane (Figure 4D) intensity profiles for DPEPO:4,6-PhPMAF and DPEPO:2,6-PhPMAF films are extracted from the 2D GI-WAXD pattern. The azimuthal angle scan X-rays of (100) reflection are measured to elucidate the molecular orientation of emitter in Figure 4E. Calculated full width at half maximum values for azimuthal intensity distributions in DPEPO:4,6-PhPMAF and DPEPO:2,6-PhPMAF emitters are estimated as 27.2° and 25.3° by Gaussian model fitting. The FWHM of DPEPO:2,6-PhPMAF film is relatively narrow compared to those of DPEPO:4,6-PhPMAF film, which can improve the outcoupling efficiency in the devices as implying horizontal plane-on orientation (Kim et al., 2016; Sohn et al., 2018).

### Transient PL Spectra Properties

**Figure 5:**
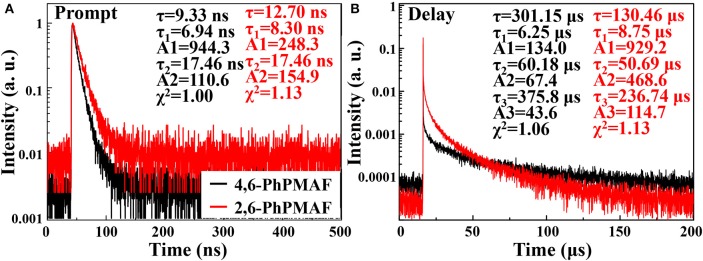
Transient photoluminescence characteristics of **(A)** prompt and **(B)** delay of 4,6-PhPMAF and 2,6-PhPMAF dopants in degassed CH_2_Cl_2_ solution and 22 wt%-doped in DPEPO (λ_ex_ = 374 nm).

Transient PL decay curve of the 4,6-PhPMAF and 2,6-PhPMAF doped DPEPO films (22%, 40 nm) are obtained to identify prompt and delayed fluorescent as shown in Figure 5. The prompt PL emission (Figure 5A) exhibited similar spectral distribution, which implied similar fluorescence emissive states, whereas the lifetimes of the delayed components of 4,6-PhPMAF and 2,6-PhPMAF doped films were 301.15 and 130.15 μs. In Figure 5B, the delayed emission of DPEPO:4,6-PhPMAF exhibited relatively weak spectra compared to those of DPEPO:2,6-PhPMAF, which implied reduced triplet exciton action, as well as suppressed Dexter energy transfer in TADF emission (Fukagawa et al., 2017; Han et al., 2019). A Δ*E*_ST_ of 4,6-PhPMAF and 2,6-PhPMAF dopants had been calculated to 0.27 and 0.17 eV, respectively. The small Δ*E*_ST_ can contribute to improved internal quantum efficiency by up-converted triplet excitons (Liu et al., 2019). However, the small Δ*E*_ST_ value of faster RISC from triplet to singlet can result in short or similar TADF lifetime (Zhang et al., 2019).

### Device Characteristics

**Figure 6:**
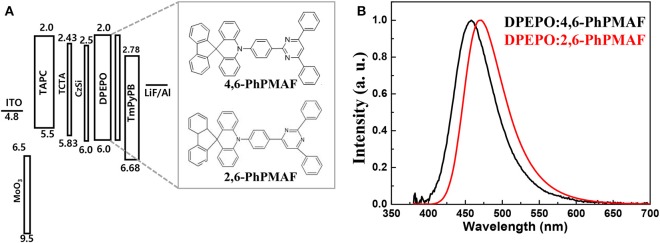
**(A)** Energy-band diagram and **(B)** normalized electroluminescence intensity of blue-emitting TADF OLEDs with DPEPO:4,6-PhPMAF and DPEPO:2,6-PhPMAF emitters.

**Figure 7:**
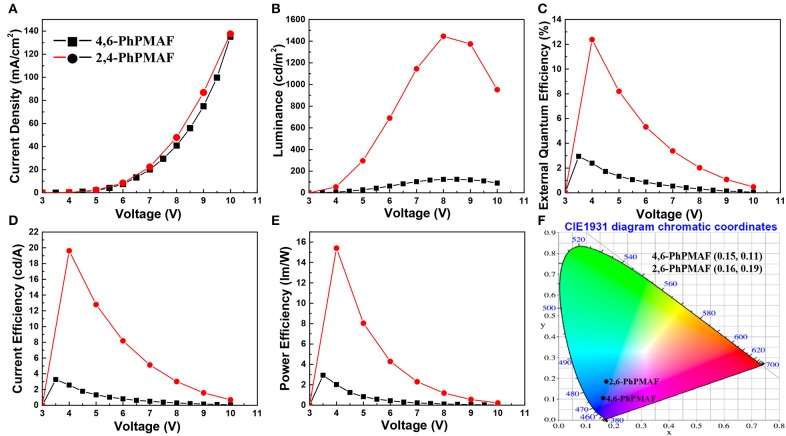
Graph of **(A)** current density, **(B)** luminance, **(C)** external quantum efficiency, **(D)** current efficiency, and **(E)** power efficiency as a function of applied voltage of devices with emitting materials and **(F)** CIE coordinate of devices based on CIE 1931.

The energy-band diagram of the device structure is shown, and the chemical structure of synthesized 4,6-PhPMAF and 2,6-PhPMAF dopants into DPEPO host material is attached in Figure 6A. For effective hole and electron balancing, we have chosen hole or electron transport layers (ETLs) carefully by considering their HOMO and LUMO energy levels and charge mobility. The HOMO and LUMO values of 4,6-PhPMAF and 2,6-PhPMAF are determined using DFT calculation and cyclic voltammetry (CV) measurement for blue emitter. The HOMO-LUMO levels of molybdenum trioxide (MoO_3_), TAPC, TCTA, DPEPO, and TmPyPB materials are obtained from Ossila Co. The normalized electroluminescent (EL) spectra are obtained for 4,6-PhPMAF and 2,6-PhPMAF doped devices (Figure 6B). The EL spectra of all the devices show solely emission peaks with any shoulder peaks, which could be contributed to good color gamut OLEDs. However, the EL spectra 4,6-PhPMAF doped device is red-shifted compared to the PL spectra, as discussed in reported 2,6-PhPMAF material (Ganesan et al., 2018). In the spectra, the EL maximum peak of the device with 4,6-PhPMAF dopant exhibit 458-nm emission, which is blue shifted compared to those of 2,6-PhPMAF dopant of 471 nm. It is caused by the different substance nitrogenous position in diphenylpyrimidine group of electron acceptor with spiroacridine-based electron donor. The current-density, luminance, external quantum efficiency, current efficiency, and power efficiency vs. applied voltage of devices are shown in Figure 7. The Commission International de l′Eclairag (CIE) coordinates of devices are displayed. The devices with DPEPO:4,6-PhPMAF emitter exhibit maximum luminescence of 213 cd/m^2^ at 8 V, external quantum efficiencies of 2.95%, current efficiency of 3.27 cd/A, and power efficiencies of 2.94 lm/W. The CIE coordinates of 4,6-PhPMAF doped device have (0.15, 0.11) at *x* and *y* axes, which is close to the deep-blue TADF OLEDs. These deep-blue emitters with CIE *y* coordinate <0.15 can be attributed the low-power consumption, as well as the color gamut when it will be applied in full-color OLEDs. The devices with DPEPO:2,6-PhPMAF emitter and TADF emitter exhibit bright luminescence with 1,445 cd/m^2^ at 8 V, maximum external quantum efficiencies of 12.38%, current efficiency of 19.6 cd/A, power efficiencies of 15.4 lm/W, and cobalt blue emitting with CIE coordinates of (0.16, 0.19). The reason that the efficiency of the device is approximately four times different is due to three reasons as follows: First, the improved device efficiencies in 2,6-PhPMAF doped device is caused by fast up-converted triplet excitons to the singlet state with relatively smaller Δ*E*_ST_ of 0.17 eV compared to 0.27 eV in 4,6-PhPMAF dopant. Second is that it is believed to be due to improved internal quantum efficiency by the relatively higher PLQY value of the 2,6-PhPMAF (29.5%) emitter than 4,6-PhPMAF (16.9%) because the device efficiency is depends on the solid-state PLQY of the emitter, as discussed in literatures (de Sá Pereira et al., 2017; Maasoumi et al., 2018). As mentioned in the azimuthal intensity distributions, finally, it can be explained because the relatively narrow FWHM of DPEPO:2,6-PhPMAF film compared with the DPEPO:4,6-PhPMAF film has relatively horizontal plane-on orientation to the substrate, resulting in improved outcoupling efficiency in the emitting layer. The synthesized acridine (D) and diphenylpyrimidine (A) based emitters can be used as high-performance deep-blue emitters in a TADF-OLEDs.

## Conclusion

The blue-emitting TADF materials with spiroacridine-based electron donor and diphenylpyrimidine group of electron acceptor with different substance nitrogenous position are successfully synthesized. The performance of reported 2,6-PhPMAF doped TADF device is over four times higher than synthesized 4,6-PhPMAF doped device. It is due to improved fast up-converted triplet excitons to the singlet state and smaller Δ*E*_ST_ as well as higher PL quantum yield in 2,6-PhPMAF than the 4,6-PhPMAF. Minor reason could be due to outcoupling efficiency in the emitting layer of the 2,6-PhPMAF-doped TADF device with relatively horizontal plane-on orientation. Despite the relatively low device efficiency with 4,6-PhPMAF, the *y* axis in CIE coordinate exhibits 0.11, which is close to the deep-blue OLEDs. This deep-blue 4,6-PhPMAF emitter could be contributed to the low power consumption, as well as good color gamut when it will be fabricated in full-color OLEDs.

## Experimental Section

### Materials

Detailed descriptions are given in the Supporting Information.

### Instruments

^1^H NMR spectra were recorded using a Bruker Avance 300 MHz FT-NMR spectrometer, ^13^C NMR were recorded using a Bruker Avance 300 MHz FT-NMR spectrometer. Chemical shifts (ppm) were reported with tetramethylsilane as an internal standard. Thermogravimetric analysis (TGA) under N_2_ gas was performed using a TA instrument 2050 thermogravimetric analyzer. Differential scanning calorimetry (DSC) under N_2_ gas was conducted using a TA instrument DSC Q10. Samples for TGA and DSC were heated at 10°C/min. UV-visible spectra were measured using a Shimadsu UV-1065PC UV-vis spectrophotometer. Photoluminescence spectra were measured using a Perkin–Elmer LS50B fluorescence spectrophotometer. The electrochemical properties of the materials were measured by CV using an Epsilon C3 in a 0.1 M solution of tetrabutyl ammonium perchlorate in acetonitrile. The topographies of 22 wt.%-doped 1 and 2 films in DPEPO had been analyzed using AFM (VEECO Dimension 3100+Nanoscope V) in non-contact mode. Grazing-incident wide-angle X-ray diffraction measurements were performed at the 9A U-SAXS beamline of the Pohang Light Source in South Korea. Grazing-incident wide-angle X-ray diffraction samples were prepared by the same processing condition with active layer casting on the <100> silicon wafer. The wavelength of X-rays was 1.12148 Å (*E* = 11.055 keV); the incidence angle was 0.12°, and the sample exposure time was 30 s. The GI-WAXD images were recorded with a 2D CCD (Rayonix MX170-HS). The diffraction angles were calibrated by a precalibrated sucrose solution (Monoclinic, P21, *a* = 10.8631 Å, *b* = 8.7044 Å, *c* = 7.7624 Å, and β = 102.938°). The sample-to-detector distance was 221 mm. The prompt and delayed fluorescence lifetimes are measured by a fluorescence lifetime spectrometer, a Quantaurus-Tau C11367-31 instrument of Hamamatsu, as measured in literature (Han et al., 2019).

### Device Fabrication and Measurements

For comparing dopant property as well as electron and hole balancing, the devices of ITO/MoO_3_ (20%): TAPC (20 nm)/TAPC (45 nm)/TCTA (5 nm)/CzSi (2 nm)/DPEPO: 4,6-PhPMAF or DPEPO: 2,6-PhPMAF 22% (20 nm)/DPEPO (3 nm)/TmPyPB (50 nm)/lithium fluoride (LiF) (1 nm)/aluminum (Al) (120 nm) had been fabricated on ITO-coated glass substrates in a class-1000 cleanroom. An ITO-coated glass was sequentially cleaned using deionized water, acetone, and isopropyl alcohol for 15 min in an ultrasonic bath and then dried in an oven at 70°C during 1 day to remove residual organic solvents and moisture on the ITO substrate. An MoO_3_ doped 4,4′-cyclohexylidenebis[N,N-*bis*(4-methylphenyl)benzenamine] (TAPC) mixed layer was used for improve hole injection. Tris(4-carbazoyl-9-ylphenyl)amine (TCTA) was used as a hole-transport layer, as well as exciton or electron-blocking layer due to its high-lying LUMO level in the devices. 9-(4-*tert*-Butylphenyl)-3,6-*bis*(triphenylsilyl)-9H-carbazole (CzSi) with high triplet energy (3.02 eV) and wide bandgap (3.5 eV) was used for enhancing morphological and electrochemical stability. To achieve efficient TADF OLEDs, *bis*[2-(diphenylphosphino)phenyl]ether oxide (DPEPO) host material was used because it has thermal and morphological stability as well as the ETL and a hole-blocking layer with high HOMO level (~6.1 eV). As an ETL material, 1,3,5-tri(m-pyrid-3-yl-phenyl)benzene (TmPyPB) was used because of its triplet energy level with deep HOMO level (6.75 eV). The TmPyPB can be used as a co-host material with the hole transporting TCTA due to its high electron mobility. Lithium fluoride and Al were, respectively, evaporated as an interlayer and a cathode. The devices were encapsulated with glass, and then their current density, luminance, and efficiencies vs. driving voltages of the devices were measured using a Keithley 236 and a CS-1000 (Konica Minolta Co.) system.

## Data Availability Statement

All datasets generated for this study are included in the article/Supplementary Material.

## Author Contributions

SS has written a manuscript and contributed the optoelectrical analysis and the fabrication of the OLEDs. MH have written a synthetic part of manuscript and characterize the synthesized materials. Y-HK synthesized the emitting materials. JP contributed to the computational analysis. HA analyzed the 2D GI-WAXD images using scattered X-ray beam. SJ and S-KK have made a substantial and intellectual contribution to the work. Y-HK proposed the idea of this manuscript and analyzed the experiment results.

## Conflict of Interest

The authors declare that the research was conducted in the absence of any commercial or financial relationships that could be construed as a potential conflict of interest.
