# Supplementary material for: High-Efficiency Diphenylpyrimidine Derivatives Blue Thermally Activated Delayed Fluorescence Organic Light-Emitting Diodes
Source: Front Chem. 2020 May 14;8:356. doi: 10.3389/fchem.2020.00356 (PMC7240071; doi:10.3389/fchem.2020.00356)
Supplement: Supplementary file 1 [file Data_Sheet_1.pdf]

Supplementary Material

1 Synthesis of molecules

1.1. Materials

All reagents were purchased from Aldrich and TCI. Solvents were dried by using standard procedures.

1.2. Instruments

^{1}H-NMR and ^{13}C-NMR spectra were recorded on a Bruker 300 and Avance 500 spectrometer. A Jeol JMS-700 mass spectrometer was used to obtain the mass spectra of the samples.

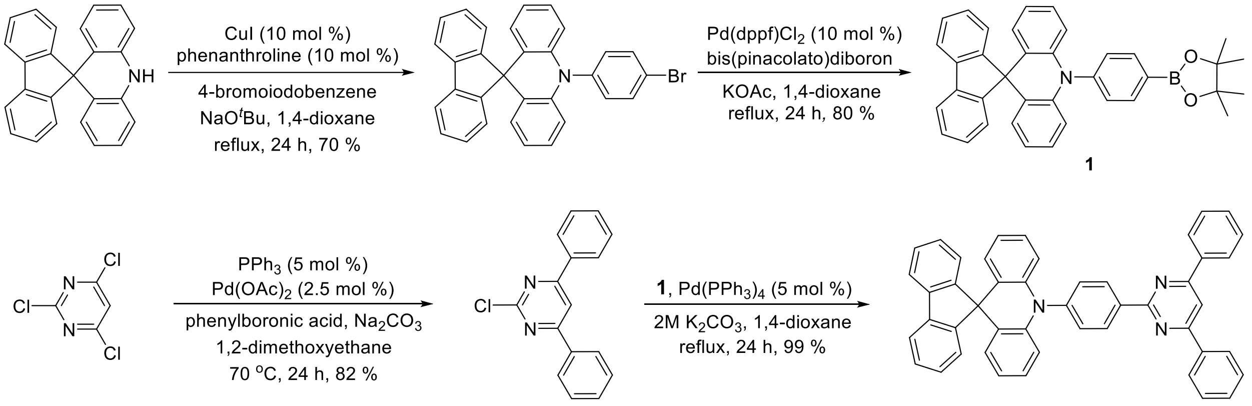

Scheme 1. Synthetic route of 4,6-PhMAF.

10-(4-bromophenyl)-10H-spiro[acridine-9,9'-fluorene To a solution of 10*H*-spiro[acridine-9,9'-fluorene] (6.0 g, 18.1 mmol) in 1,4-dioxane (250 mL) was added sodium *tert*-butoxide (36.2 mmol) at room temperature under N2 gas. After 30 min the reaction mixture was treated with copper (I) iodide (1.8 mmol), 1,10-phenanthroline (1.8 mmol), and 4-bromoiodobenzene (19.9 mmol). And then the reaction was stirred under reflux condition for 24 h. When the reaction was completed, the reflux apparatus was removed cooling the solution to room temperature. The mixture was filtered through a pad of Celite and concentrated. Purification by column chromatography on silica gel afforded the desired product as a white solid (6.16 g, 70 %). ^{1}H NMR (300 MHz, CD_2Cl_2) δ 7.87 (t, J = 7.5 Hz, 4H), 7.41 (m, 6H), 7.29 (td, J_1 = 7.4 Hz, J_2 = 1.1 Hz, 2H), 6.95 (td, J_1 = 7.8 Hz, J_2 = 1.6 Hz, 2H), 6.58 (td, J_1 = 7.5 Hz, J_2 = 1.1 Hz, 2H), 6.38 (d, J = 8.0 Hz, 4H) ppm; ^{13}C NMR (75 MHz, CD_2Cl_2) δ 156.0, 140.6, 139.7, 138.8, 134.0, 132.7, 127.9, 127.3, 126.9, 125.0, 120.2, 119.6, 114.2, 56.3 ppm.

10-(4-(4,4,5,5-tetramethyl-1,3,2-dioxaborolan-2-yl)phenyl)-10H-spiro[acridine-9,9'-fluorene] To a solution of 10-(4-bromophenyl)-10*H*-spiro[acridine-9,9'-fluorene] (5.0 g, 10.3 mmol) in 1,4-dioxane (30 mL) were added (1,1'-bis(diphenylphosphino)ferrocene)palladium(II) dichloride (1.0 mmol), potassium acetate (30.8 mmol), and pinacol borate (12.3 mmol) under N_2 gas. The reaction mixture was refluxed for 24 h. Distilled water (50 mL) was added to the reaction at room temperature, and extraction with methylene chloride was performed. The organic layers were collected and dried over anhydrous $MgSO_4$. After filtration and concentration, the crude product was loaded through a column chromatography on silica gel to afford the product as a white solid

(4.4 g, 80 %). ^{1}H NMR (300 MHz, CD_2Cl_2) δ 8.15 (d, J = 8.0 Hz, 2H), 7.86 (d, J = 7.5 Hz, 2H), 7.54 (d, J = 8.0 Hz, 2H), 7.42 (m, 4H), 7.30 (m, 2H), 6.92 (td, J_1 = 7.6 Hz, J_2 = 1.4 Hz, 2H), 6.57 (td, J_1 = 7.5 Hz, J_2 = 0.9 Hz, 2H), 6.38 (m, 4H), 1.43(s, 12H) ppm; ^{13}C NMR (75 MHz, CD_2Cl_2) δ 156.1, 143.3, 140.8, 138.8, 137.0, 130.1, 127.9, 127.3, 127.0, 126.8, 125.1, 120.0, 119.6, 114.3, 83.8, 56.4, 31.2, 24.3, 22.2, 13.5 ppm.

2-chloro-4,6-diphenylpyrimidine To a solution of 2,4,6-trichloropyrimidine (20 g, 109.0 mmol) in 1,2-dimethoxyethane (350 mL) was added phenylboronic acid (218.1 mmol) and sodium carbonate (697.9 mmol). The reaction mixture was treated with palladium acetate (2.5 mol %) and triphenylphosphine (5 mol %, and then the reaction was heated to 70 °C for 24 h. General extraction with methylene chloride was performed and the organic layers were combined and dried over anhydrous $MgSO_4$. After filtration and concentration, the crude product was purified by column chromatography on silica gel affording a white solid (23.7 g, 82 %). The structure of obtained product was identified through comparison of the known data in literature (Schomaker & Delia, 2001).

10-(4-(4,6-diphenylpyrimidin-2-yl)phenyl)-10H-spiro[acridine-9,9'-fluorene] (4,6-DPSF) The generated borane, 10-(4-(4,4,5,5-tetramethyl-1,3,2-dioxaborolan-2-yl)phenyl)-10*H*-spiro[acridine-9,9'-fluorene] (1.5 g, 2.8 mmol) and 2-chloro-4,6-diphenylpyrimidine (900 mg, 3.4 mmol) were dissolved in 1,4-dioxane (20 mL) under N2 gas. The mixture was treated with 2M of aqueous potassium carbonate (10 mmol) and tetrakis(triphenylphosphine)palladium(0) (5 mol %), and then refluxed for 24 h. Addition of distilled water (100 mL) occurred solid precipitation which was the desired final product. The generated stuff was filtered and collected as a white solid (1.79 g, 99 %) 1H NMR (300 MHz, $CDCl_3$) δ 9.05 (d, J = 8.4 Hz, 2H), 8.35 (m, 4H), 8.11 (s, 1H), 7.81 (d, J = 7.5 Hz, 2H), 7.68 (d, J = 8.4 Hz, 2H), 7.61 (m, 6H), 7.48 (d, J = 7.5 Hz, 2H), 7.39 (t, J = 7.4 Hz, 2H), 7.28 (t, J = 7.4 Hz), 6.93 (td, J_1 = 7.7 Hz, J_2 = 1.4 Hz, 2H), 6.59 (t, J = 7.4 Hz, 2H), 6.47 (m, 4H) ppm; ^{13}C NMR (75 MHz, $CDCl_3$) δ 165.1, 164.0, 156.7, 143.4, 141.2, 139.3, 138.5, 137.4, 131.40, 131.38, 131.1, 129.1, 128.5, 127.9, 127.7, 127.4, 127.3, 125.9, 124.9, 120.7, 120.0, 114.8, 110.7, 56.9 ppm; MS (FAB): m/z calcd for $C_{47}H_{32}Ns_3$ ($[M+H]^+$); 638, found 638.

10-(4-(2,6-diphenylpyrimidin-4-yl)phenyl)-10H-spiro[acridine-9,9'-fluorene] According to the synthetic procedure shown in scheme 1, but only 4-chloro-2,6-diphenylpyrimidine instead of 2-chloro-4,6-diphenylpyrimidine was used for preparation of the pyrimidine isomer. The spectroscopic and spectrometric results of the product was in all agreement with the already reported data (Ganesan et al., 2018).

2 Computations of the rates of intersystem crossing (ISC) and reverse intersystem crossing (RISC)

We computed the rates of intersystem crossing (k_{ISC}) and reverse intersystem crossing (k_{RSC}) based on the Marcus theory of electron transfer (Marcus, 1993):

$$k_{ISC} = \frac{2\pi}{h}|V_{SOC}|^2 \left[\frac{1}{\sqrt{4\pi\lambda RT}} exp\left(-\frac{(-\Delta E_{ST}+\lambda)^2}{4\pi\lambda RT}\right)\right]$$

$$\text{and } k_{RISC} = \frac{2\pi}{h}|V_{SOC}|^2 \left[\frac{1}{\sqrt{4\pi\lambda RT}} exp\left(-\frac{(\Delta E_{ST}+\lambda)^2}{4\pi\lambda RT}\right)\right]$$

, where V_{soc} is the strength of spin–orbit coupling that mediates the intersystem crossing processes, $\hbar$ is the reduced Planck's constant, R is the Gas constant, T is the temperature, ΔE_{ST} is the electronic

energy difference of the singlet and the triplet state, λ is the reorganization energy. Here, k_{ISC} refers to the rate of transition from the singlet excited state (S_1) to the triplet excited state (T_1) and k_{RISC} indicates the rate of reverse process.

Table S2 summarizes details of the computations of k_{ISC} and k_{RISC}. For the rates of ISC, the reorganization energies (λ) were computed as the electronic energy of S_1 computed at the optimized T_1 geometry. The spin–orbit coupling elements (V_{soc}) were measured at the optimized T_1 geometry as well. Likewise, for the rates of RISC, the reorganization energies (λ) were calculated as the electronic energy of T_1 computed at the optimized S_1 geometry. Again, the spin–orbit coupling elements (V_{soc}) were computed at the optimized S_1 geometry for the rates of reverse ISC. We used Q-Chem 5.0 software suite (Shao et al., 2015) for the identifications of the excited state geometries based on time-dependent density functional theory (TD-DFT) (Li & Tong, 1986) and calculations of the corresponding electronic energies and the spin–orbit coupling elements. (Samanta, Kim, Coropceanu, & Brédas, 2017) A range-separated hybrid density functional theory, namely, CAM–B3LYP in conjunction with a double–zeta quality Gaussian basis set was adopted to describe the nature of charge–transfer states. (Francl et al., 1982; Yanai, Tew, & Handy, 2004)

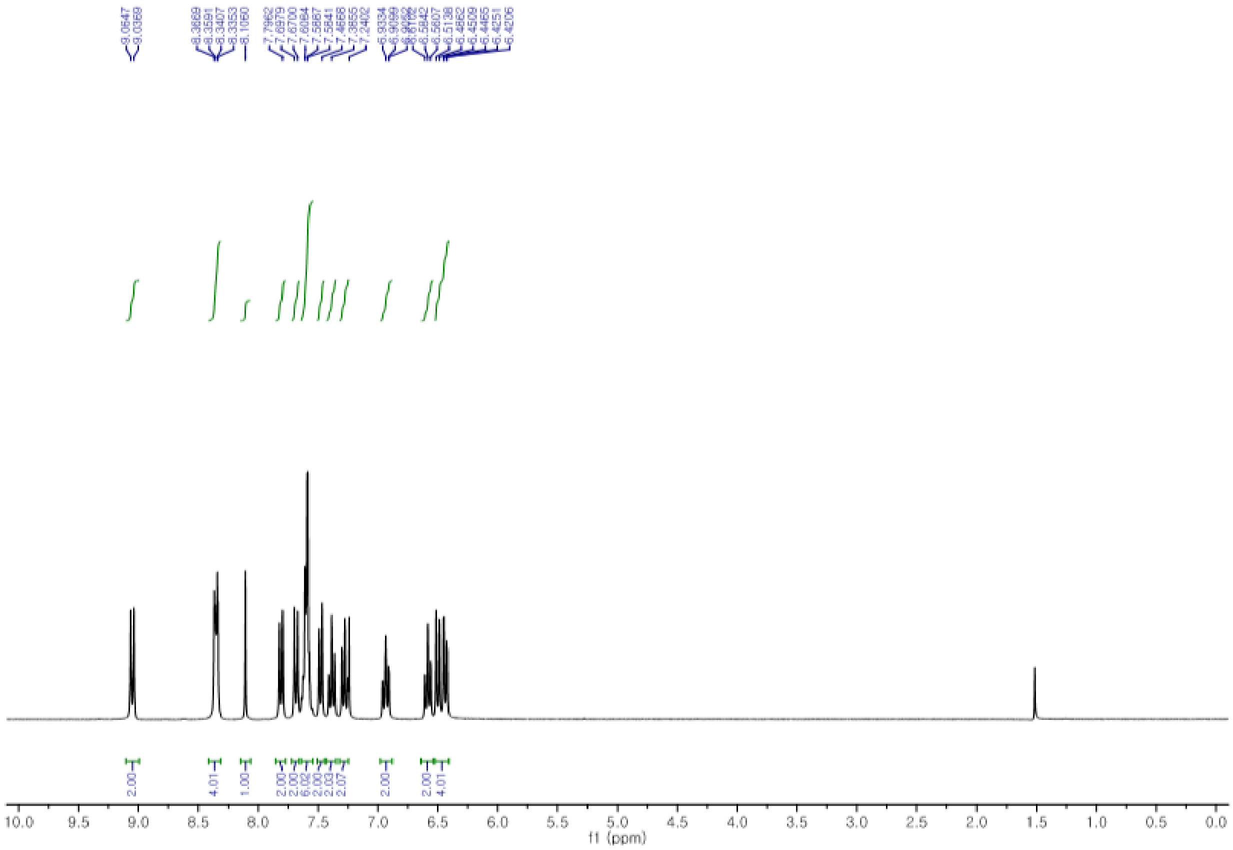

Supplementary Figure 1. ^{1}H NMR spectroscopy of 10-(4-(4,6-diphenylpyrimidin-2-yl)phenyl)-10*H*-spiro[acridine-9,9'-fluorene].

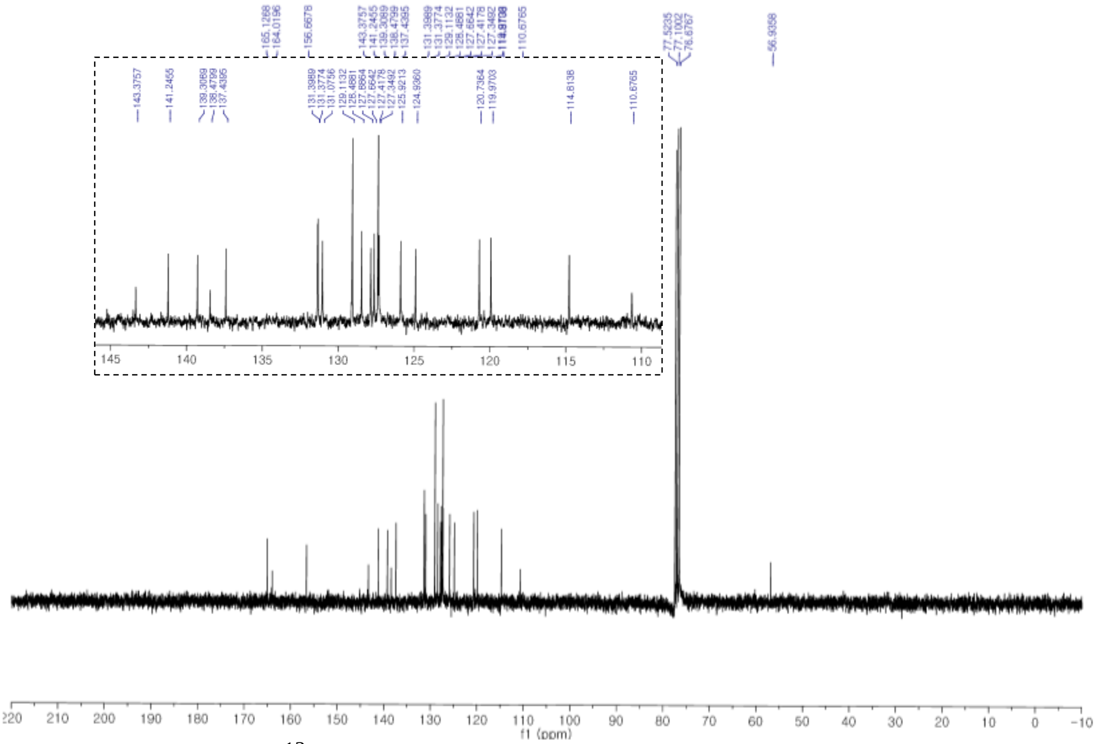

Supplementary Figure 2. ^{13}C NMR spectroscopy of 10-(4-(4,6-diphenylpyrimidin-2-yl)phenyl)-10*H*-spiro[acridine-9,9'-fluorene].

4,6–PhPMAF	S_0 (0.00)	S_1 (3.65)	T_1 (3.38)
	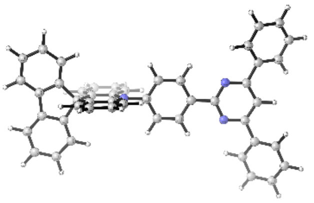	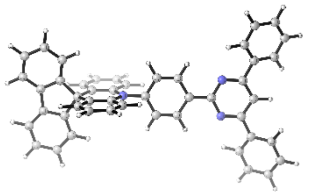	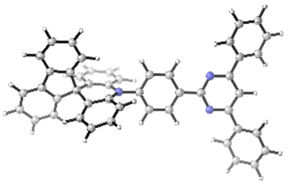
2,6–PhPMAF	S_0 (0.00)	S_1 (3.55)	T_1 (3.38)
	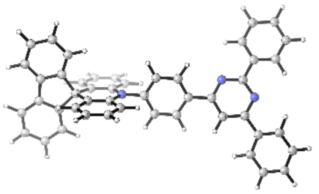	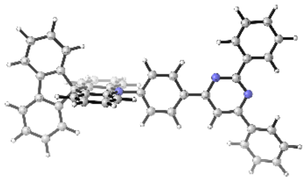	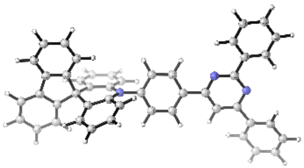

Supplementary Figure 3. Optimized geometries of 4,6-PhPMAF and 2,6-PhPMAF. The electronic energies relative to the optimized ground state geometries are shown in parentheses [eV].

4,6–PhPMAF	HOMO (-6.18)	LUMO (-0.57)
	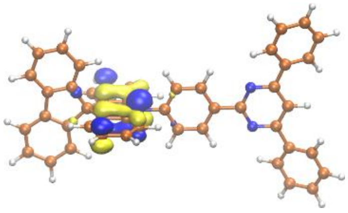	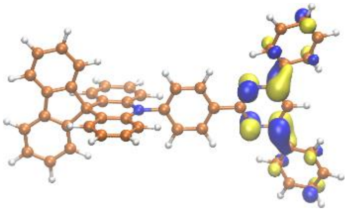
2,6–PhPMAF	HOMO (-6.31)	LUMO (-0.60)
	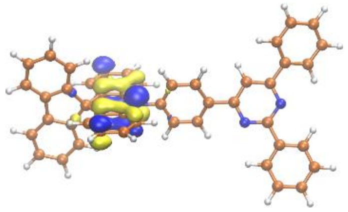	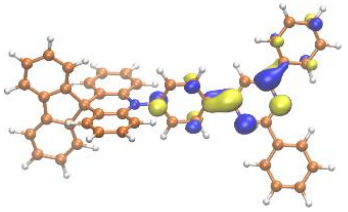

Supplementary Figure 4. HOMO and LUMO levels in the optimized ground state geometries of 4,6-PhPMAF and 2,6-PhPMAF. Orbital lobes are shown at isodensity of 0.05. The electronic energies in eV are shown in parentheses.

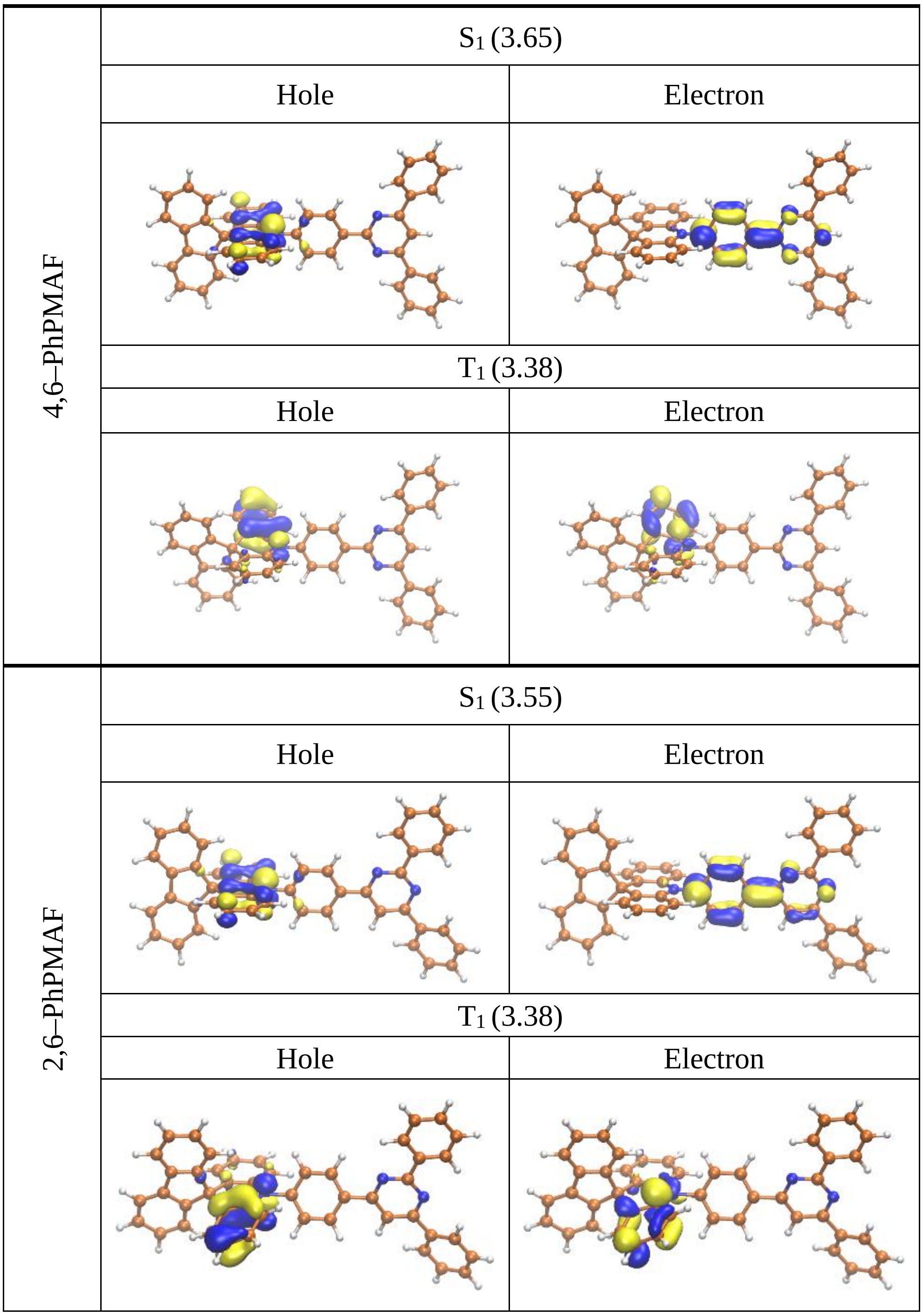

Supplementary Figure 5. Natural transition orbital (NTO) analysis of the singlet and triplet excited state geometries of 4,6-PhPMAF and 2,6-PhPMAF. Orbital lobes are shown at isodensity of 0.05. The electronic energies relative to the optimized ground state geometries are shown in parentheses [eV].

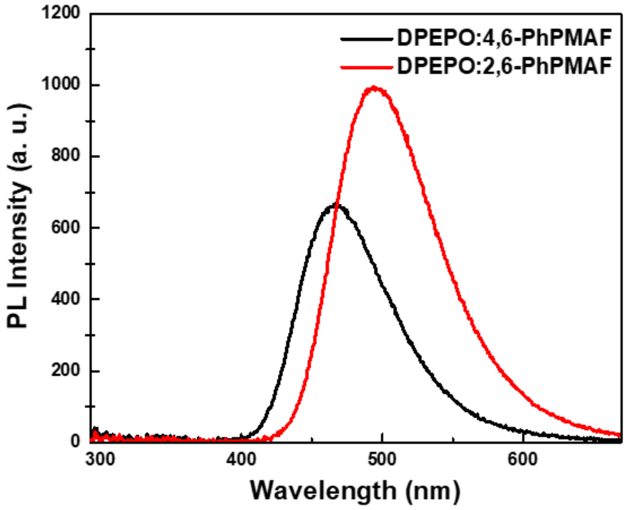

Supplementary Figure 6. PL spectra of DPEPO:4,6-PhPMAF and DPEPO:2,6-PhPMAF at low temperature 77 K (excitation at 300 nm for the PL).

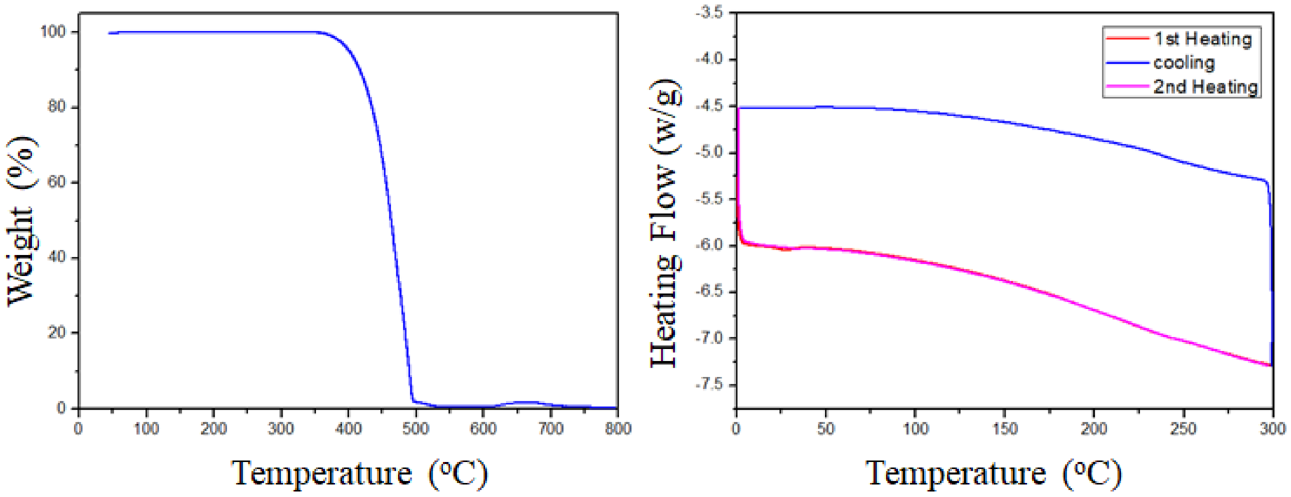

Supplementary Figure 7. TGA and DSC thermograms of 4,6-PhPMAF.

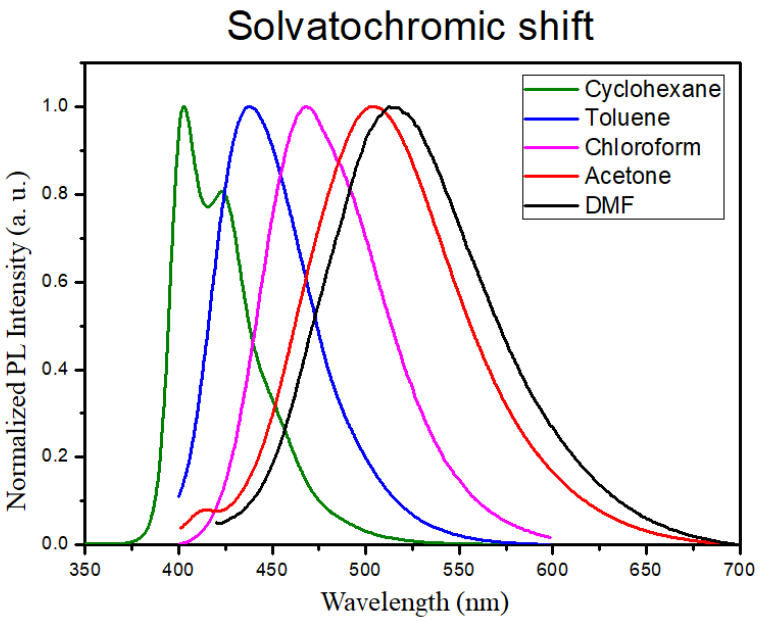

Supplementary Figure 8. The solution PL spectra of 4,6-PhPMAF in cyclohexane, chloroform, acetone, and DMF.

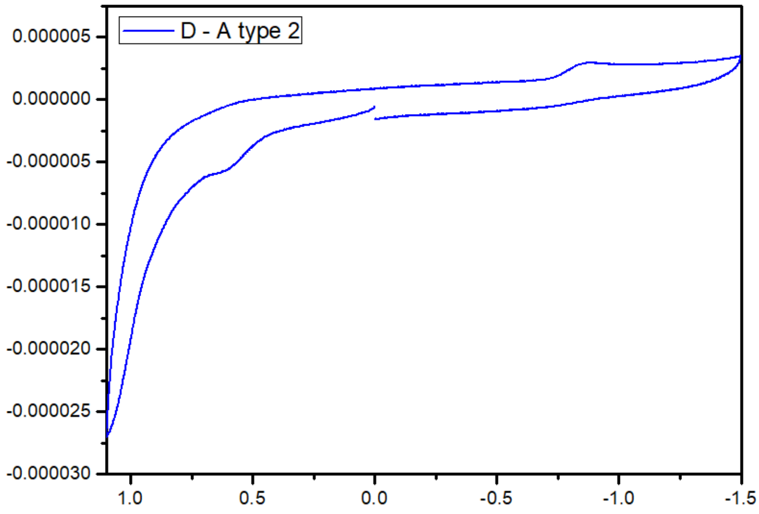

Supplementary Figure 9. The CV curve of 4,6-PhPMAF.

Supplementary Table 1. Computed oscillator strength and dipole moments of the first singlet excited state (S_1) of 4,6-PhPMAF and 2,6-PhPMAF.

Emitter	Oscillator strength [f_{OSC}]	Dipole moment [Debye·Å)
4,6–PhPMAF	0.002640	18.38
2,6–PhPMAF	0.004138	22.15

Supplementary Table 2. Details of the calculations of the rates of ISC and RISC for 4,6-PhPMAF and 2,6-PhPMAF.

Emitter	$E(S_1)$ [eV]	$E(T_1)$ [eV]	V_{SOC} [cm^{-1}]	ΔE_{ST} [eV]	PLQY (%)	λ_{ISC} [eV]	k_{ISC} [s^{-1}]	V_{SOC}	λ_{RISC}	k_{RISC}
4,6–PhPMAF	-53682.16	-53682.44	1.511	0.27	16.9	0.69	3.3×10^8	1.786	0.39	3.9×10^7
2,6–PhPMAF	-53682.25	-53682.42	1.460	0.17	29.5	0.77	1.5×10^8	1.769	0.40	1.0×10^8

Supplementary Table 3. Optical, electrochemical, and thermal properties of 4,6-PhPMAF.

UV(nm) solution	PL (nm)	FWHM	E_g (eV)	HOMO (eV)	LUMO (eV)	T_d (°C)	T_g (°C)	T_m (°C)
309, 368	438	58	3.03	-5.30	-2.27	400	-	-

References

Francl, M. M., Pietro, W. J., Hehre, W. J., Binkley, J. S., Gordon, M. S., DeFrees, D. J., & Pople, J. A. (1982). Self-consistent molecular orbital methods. XXIII. A polarization-type basis set for second-row elements. *The Journal of Chemical Physics*, *77*(7), 3654–3665. https://doi.org/10.1063/1.444267

Ganesan, P., Chen, D. G., Liao, J. L., Li, W. C., Lai, Y. N., Luo, Di., … Chi, Y. (2018). Isomeric spiro-[acridine-9,9′-fluorene]-2,6-dipyridylpyrimidine based TADF emitters: Insights into photophysical behaviors and OLED performances. *Journal of Materials Chemistry C*, *6*(37), 10088–10100. https://doi.org/10.1039/c8tc03645d

Li, T. C., & Tong, P. Q. (1986). Time-dependent density-functional theory for multicomponent systems. *Physical Review A*, *34*(1), 529–532. https://doi.org/10.1103/PhysRevA.34.529

Marcus, R. A. (1993). Electron transfer reactions in chemistry. Theory and experiment. *Reviews of Modern Physics*, *65*(3), 599–610. https://doi.org/10.1103/RevModPhys.65.599

Samanta, P. K., Kim, D., Coropceanu, V., & Brédas, J. L. (2017). Up-Conversion Intersystem Crossing Rates in Organic Emitters for Thermally Activated Delayed Fluorescence: Impact of the Nature of Singlet vs Triplet Excited States. *Journal of the American Chemical Society*, *139*(11), 4042–4051. https://doi.org/10.1021/jacs.6b12124

Schomaker, J. M., & Delia, T. J. (2001). Arylation of halogenated pyrimidines via a suzuki coupling reaction. *Journal of Organic Chemistry*, *66*(21), 7125–7128. https://doi.org/10.1021/jo010573+

Shao, Y., Gan, Z., Epifanovsky, E., Gilbert, A. T. B., Wormit, M., Kussmann, J., … Head-Gordon, M. (2015). Advances in molecular quantum chemistry contained in the Q-Chem 4 program package. *Molecular Physics*, *113*(2), 184–215. https://doi.org/10.1080/00268976.2014.952696

Yanai, T., Tew, D. P., & Handy, N. C. (2004). A new hybrid exchange-correlation functional using the Coulomb-attenuating method (CAM-B3LYP). *Chemical Physics Letters*, *393*(1–3), 51–57. https://doi.org/10.1016/j.cplett.2004.06.011
